# Supplementary material for: DEPEND study protocol: early detection of patients with pancreatic cancer – a pilot study to evaluate the utility of faecal elastase-1 and 13C-mixed triglyceride breath test as screening tools in high-risk individuals
Source: BMJ Open. 2022 Feb 25;12(2):e057271. doi: 10.1136/bmjopen-2021-057271 (PMC8883257; doi:10.1136/bmjopen-2021-057271)
Supplement: Author's manuscript [file bmjopen-2021-057271.draft_revisions.pdf]

BMJ Open is committed to open peer review. As part of this commitment we make the peer review history of every article we publish publicly available.

When an article is published we post the peer reviewers' comments and the authors' responses online. We also post the versions of the paper that were used during peer review. These are the versions that the peer review comments apply to.

The versions of the paper that follow are the versions that were submitted during the peer review process. They are not the versions of record or the final published versions. They should not be cited or distributed as the published version of this manuscript.

BMJ Open is an open access journal and the full, final, typeset and author-corrected version of record of the manuscript is available on our site with no access controls, subscription charges or pay-per-view fees (http://bmjopen.bmj.com).

If you have any questions on BMJ Open's open peer review process please email info.bmjopen@bmj.com

BMJ Open

DEPEND study protocol: Early detection of patients with pancreatic cancer: a pilot study to evaluate the utility of faecal elastase-1 and 13C-mixed triglyceride breath test as screening tools in high-risk individuals.

Journal:	*BMJ Open*
Manuscript ID	bmjopen-2021-057271
Article Type:	Protocol
Date Submitted by the Author:	11-Sep-2021

Complete List of Authors:	Afolabi, Paul; University of Southampton Faculty of Medicine; NIHR Southampton Biomedical Research Centre, Southampton Centre for Biomedical Research Mass Spectrometry<br>McDonnell, Declan; University of Southampton Faculty of Medicine; NIHR Southampton Biomedical Research Centre, Southampton Centre for Biomedical Research<br>Byrne, C. D.; University of Southampton Faculty of Medicine; NIHR Southampton Biomedical Research Centre, Southampton Centre for Biomedical Research<br>Wilding, Sam; University Hospital Southampton NHS Foundation Trust, Southampton Clinical Trials Unit.<br>Goss, Victoria; University Hospital Southampton NHS Foundation Trust, Southampton Clinical Trials Unit<br>Walters, Jocelyn; University Hospital Southampton NHS Foundation Trust, Southampton Clinical Trials Unit.<br>Hamady, Zaed; University Hospital Southampton NHS Foundation Trust; NIHR Southampton Biomedical Research Centre, Southampton Centre for Biomedical Research

Keywords:	Endocrine tumours < DIABETES & ENDOCRINOLOGY, Gastrointestinal tumours < GASTROENTEROLOGY, Pancreatic surgery < SURGERY, Biochemistry < NATURAL SCIENCE DISCIPLINES, Pancreatic disease < GASTROENTEROLOGY

SCHOLARONE™
Manuscripts

DEPEND study protocol: Early detection of patients with pancreatic cancer: a pilot study to evaluate the utility of faecal elastase-1 and ^{13}C-mixed triglyceride breath test as screening tools in high-risk individuals.

Paul R Afolabi [1,2], Declan McDonnell [1,2,3], Christopher D Byrne [1,2,3] , Sam Wilding [4], Victoria M Goss [4], Jocelyn Walters [4], Zaed Z Hamady [1,2,3].

Correspondence to Mr Zaed Hamady; Z.Hamady@soton.ac.uk

For peer review only

Abstract

Introduction Pancreatic cancer (PC) is the fifth leading cause of cancer related death in the UK. The incidence of PC is increasing, with little or no improvement in overall survival and the best chance for long-term survival in patients with PC relies on early detection and surgical resection. In this study, we propose the use of a diagnostic algorithm that combines tests of pancreatic exocrine function (Faecal elastase-1 test (FE-1) and the ^{13}C-mixed triglyceride (^{13}C-MTG) breath test) to identify patients with PC that urgently needs imaging studies.

Methods and analysis This prospective pilot (proof of concept) study will be carried out on 25 patients with resectable PC, 10 patients with chronic pancreatitis and 25 healthy volunteers. This study will construct a predictive algorithm for PC, utilising two tests of pancreatic exocrine function, FE-1 test and the ^{13}C-MTG breath test. Continuous flow isotope ratio mass spectrometry in the ^{13}C-MTG breath test will be used to analyse enriched $^{13}CO_2$ in exhaled breath samples. The additional predictive benefit of other potential biomarkers of PC will also be considered. Potential biomarkers of PC showing abilities to discriminate between patients with PC from healthy subjects or patients with chronic pancreatitis will be selected by metabolomic analysis.

Ethics and dissemination The study was approved by the North of Scotland Research and Ethics Committee on 1 October 2020 (reference: 20/NS/0105, favourable opinion granted). The results will be disseminated in presentations at academic national/international conferences and publication in peer-review journals.

For peer review only

Strength and limitations of this study

- This pilot study is aiming to be the first study to construct a predictive algorithm for the early detection of pancreatic cancer (PC) based on tests of pancreatic exocrine function.
- PC patients with potentially curative pancreatic resection will be recruited in this study.
- We expect poor apparent ability for the algorithm to discriminate between PC and chronic pancreatitis patients due to limited sample size.
- Participants' return of faecal specimens may be limited by embarrassment, hesitancy or inability of the study participants to provide a sample.

For peer review only

INTRODUCTION

Pancreatic cancer (PC) is the fifth leading cause of cancer related death in the UK. The UK has one of the worst survival rates in Europe, with an average life expectancy at diagnosis of just 4–6 months and 20% survival at 1 year. In addition, only 3% of people survive for 5 years or longer and this figure has not improved four decades[1]**.** Since complete surgical resection remains the only potentially curative option for PC, it is crucial to identify PC at an early stage, when it is still resectable. Unfortunately, only 10–20% of patients with PC are diagnosed at a stage when curative surgery remains an option[2].

The early symptoms of PC are usually vague and include weight loss, upper abdominal pain, backache, fatigue, diarrhoea and nausea. In 93% of patients, these symptoms may have been present for up to two years before diagnosis of PC [3]. Recent studies show that around 40% of patients diagnosed with PC require three or more visits to their GP before they are referred to a specialist [4 5] thereby leading to a delay in the diagnosis. There is also an increased risk of developing PC in those with new-onset diabetes mellitus (NODM) i.e. less than three years since diagnosis [6] and as many as 25% of PC cases are believed to present with NODM [7].

Recent survey of GPs commissioned by Pancreatic Cancer Action [8] suggested that the delay between not having access to a reliable diagnostic referral pathway and a confirmed diagnosis of PC is a major barrier to the early detection of PC. As the symptoms of PC are often non-specific and unlikely to prompt further clinical investigation, there is an urgent need to have a reliable and cheap screening test that can be used to identify high-risk individuals who require urgent imaging studies such as CT scan to confirm or exclude the

For peer review only

presence of a pancreatic mass [9]. Having such a test with reliable sensitivity and negative predictive value (NPV), would enable the GP to select patients for urgent referral to further investigations of PC. A high NPV value would enable the GP to reassure the patient that they did not need further imaging studies. Our innovative approach is to use tests of pancreatic exocrine function combined as a screening test in patients presenting with the vague symptoms that may be associated with PC.

Although pancreatic exocrine insufficiency (PEI) is a well-known complication of pancreatic cancer [10 11], its presence is frequently overlooked in patients with advanced PC resulting in a decreased quality of life (QoL), malnutrition and morbidity [12]. Currently, the diagnostic tests of pancreatic exocrine function such as faecal elastase-1 (FE-1) or the ^{13}C-mixed triglyceride (^{13}C-MTG) breath test, may be used to detect PEI in patients after pancreatic surgery and chronic pancreatitis [13-16].

FE-1 test is a simple indirect and non-invasive method for assessing pancreatic secretion, which measures faecal concentrations of elastase-1, a proteolytic enzyme produced exclusively by pancreatic acinar cells, which binds to bile salts and passes through the gut with minimal degradation. FE-1 level correlate well with output of other pancreatic enzymes, such as amylase, lipase and trypsin [17 18] and have been shown to detect PEI in patients with advanced PC and following pancreatic surgery [14-16].

The ^{13}C-MTG breath test assesses pancreatic lipase, it involves the ingestion of a ^{13}C-labeled mixed triglyceride (1,3-distearoyl, 2- (carboxyl-^{13}C)-octanoyl glycerol) mixed with a test meal. The ^{13}C-MTG contains a ^{13}C-labeled medium-chain fatty acid (^{13}C-octanoate) at the Sn-

For peer review only

2 position, and two long-chain fatty acids (stearic acid) at the Sn-1 and Sn-3 positions of the glycerol backbone of the triacylglycerol. The ingested ^{13}C-MTG then undergoes the lipolysis of the two-long chain fatty acids from ^{13}C-MTG by pancreatic lipase to produce free fatty acids (stearic acid and ^{13}C-octanoate) and monoacylglycerol. After the intestinal absorption, the ^{13}C-free fatty acids are oxidised in the liver producing $^{13}CO_2$, which is then released on breath. Therefore, the $^{13}CO_2$ concentration in breath reflects pancreatic lipase activity. The ^{13}C-MTG breath test can be an alternative or complementary to the FE-1 test for PEI. The ^{13}C-MTG breath test has been validated in patients with chronic pancreatitis [19] and patients with pancreatic cancer following surgical resection of the cancer [20 21], but not prior to surgery in patients with resectable PC.

In this study, we will validate FE-1 test and ^{13}C-MTG breath test in patients with resectable PC (at an early stage) and test their performance in discriminating between patients with PC and healthy subjects. These tests can provide a non-invasive risk stratification tool to identify patients at risk of having PC when they present with NODM, weight loss or vague upper GI symptoms. Furthermore, this will provide a rational approach to patients identified to have PEI to be selected to have pancreatic imaging such as computed tomography (CT) scan to confirm the presence of PC and having a pancreatic resection earlier (Figure 1). In addition, we will also be identifying other potential biomarkers of PC through unbiased multiomics analysis of blood samples (proteomics and metabolomics). The resulting data will be compared to the healthy control group and patients with chronic pancreatitis aiming to identify signals that are distinct to PC.

For peer review only

METHODS AND ANALYSIS

Main centre

NIHR Southampton Biomedical Research Centre, Southampton Centre for Biomedical Research, Southampton General Hospital, Southampton, UK.

Dates of the study

From 16th March 2021 to 16th March 2022.

Design

This is a single cross-sectional pilot study of patients with PC, chronic pancreatitis and healthy controls, matched for age and sex. The primary objective is to determine if ^{13}C-mixed triglyceride (^{13}C-MTG) breath test and faecal elastase-1 (FE-1) concentrations combined can discriminate between the groups. Patients with PC will be recruited at the pre-operative stage within two weeks of initial diagnosis. The secondary objective of this study is the analysis of multiomic (proteomic and metabolomic) profiles and comparison between the groups.

Inclusion criteria

Male and female participants aged from 30-85 within the Wessex pancreatic cancer catchment area will be suitable for inclusion. In the cancer cohort, patients with a diagnosis of resectable pancreatic ductal adenocarcinoma (PDAC) (stage 1-2) will be included. Patients with chronic pancreatitis and healthy subjects will also be recruited as control groups and matched for age and sex to the cases of PDAC.

Exclusion criteria

Patients and controls with autoimmune or other chronic inflammatory conditions, or those who have previously had pancreatic disease or liver dysfunction will be excluded.

For peer review only

Intervention(s) or method

Each participant will be given a participant information sheet explaining the research and will sign an informed consent form. Participants are required to fast overnight for at least 12 hours prior to undergoing the ^{13}C-mixed triglyceride (^{13}C-MTG). At the Clinical Research Facility (CRF), full infection control precautions (related to COVID-19 pandemic) will be adhered to throughout the visit. Participants will have their temperature checked on arrival to ensure they are not pyrexial as part of screening for Covid-19, which will exclude them from the study. Anthropometric measurements (height, weight and waist circumference) of the participants will be recorded. Baseline blood samples will be taken prior to undergoing the ^{13}C-MTG breath test. The data collected will include the following: Full blood count (FBC), Urea and Electrolyte blood test (U&E), Liver function tests (AST, ALT, ALP, albumin and GGT) /clotting studies, glycated hemoglobulin (HbA1c %), Lipid profile, Vitamins and micronutrients, Hyaluronic acid, Procollagen III amino terminal peptide (PIINP) and Tissue inhibitor of metalloproteinase-1 (TIMP-1). These tests will be analysed immediately after the blood has been drawn from the participants by the chemical pathology service laboratory at Southampton General Hospital. Further blood samples will be processed and stored for multiomics analysis. Participants will also be asked to provide spot stool samples for faecal elastase-1 (FE-1) concentrations by using a commercially available ELISA kit. Results of the FE-1 test will be expressed as µg/g of stool.

For peer review only

^{13}C-MTG breath test protocol

Each participant will be asked to provide 2 baseline breath samples by blowing into 2 × 10ml exetainer tubes through a straw while wearing a facemask. After that, participants will be given an oral dose of 250mg of 2-[^{13}C]-octanoyl-1,3-distearin (^{13}C-MTG -Cambridge Isotope Laboratories, Andover, MA, USA) together with a solid test meal consisting of a crispbread or other gluten-free alternative, and 200ml of water. Post-prandial breath samples will be then collected every 30mins for 4 hours. No food or drinks except for water will be allowed during the duration of the breath test study. The collected breath samples will be analysed for the ratio of $^{13}CO_2$ to $^{12}CO_2$ using a Continuous Flow Isotope Ratio Mass Spectrometer (CF-IRMS SERCON Ltd, Crewe, UK) at the Southampton Centre for Biomedical Research. The increase in $^{13}CO_2$ content with regard to the baseline value of the initial breath sample is expressed as atom % excess and we will express the results of the ^{13}C-MTG breath test as the cumulative percentage of ^{13}C-label recovered on breath as $^{13}CO_2$ over 4 hours (cPDR over 4 hours).

Primary Outcome: Prediction of PC using a logistic model with measurement of FE-1 concentration expressed as µg/g of stool and ^{13}C-MTG breath test expressed as cPDR over 4hrs as covariates.

For peer review only

Statistical analysis and data analysis plans

Sample size calculation

The sample size calculation for the proposed study is based on a previous study assessing pancreatic exocrine function using a ^{13}C-triglyceride breath test in healthy subjects and patients with a localised pancreatic mass [13]. Using data from this study, we calculated that a sample size of 25 subjects in each group (patients with PC, patients with chronic pancreatitis and healthy control subjects) will give 100% power to detect a difference of 17.8% between patients with PC and healthy control group assuming a standard deviation of 10.5% in patients with PC at a significance level of 5% using a two-tailed test. The power calculation was carried out using IBM SPSS SamplePower version 3.

Statistical tests

Data will be presented as mean + SD, median + IQR when appropriate and 95% CI. Comparison of results of FE-1 and the ^{13}C-MTG breath test between each group will be evaluated by employing either the one way analysis of variance test (if the variables are normally distributed and parametric validity conditions are fulfilled) or the Mann Whitney U-test or Kruskal-Wallis test (if the variables are not normally distributed and non-parametric validity conditions are fulfilled). A multi-variable logistic regression analysis will be performed in order to determine the independent factors that are significantly associated with the presence of PDAC and logistic regression models will be constructed based on the identified independent factors. Furthermore, multi-variable regression analysis will be used to determine the optimal algorithm that combines the results of FE-1 test and ^{13}C-MTG breath test. We will also test whether potential biomarkers of PC identified by unbiased omics analysis improve the prediction of PDAC. A Receiver operating characteristic (ROC) curve will be constructed to evaluate the diagnostic accuracy (sensitivity, specificity,

For peer review only

positive and negative predictive value, receiver operator curve analysis) of the algorithm for PDAC. Furthermore, the optimal cut off point for the algorithm to identify patients with PC will be estimated using the Youden index. A two-sided p value of <0.05 will be considered to indicate statistical significance. All analysis will be performed using SPSS (IBM, V.25.0) or Stata (Statacorp, v16.0).

Patient and public involvement statement

Patients, healthy subjects and the public were not involved in the study design, recruitment and conduct. We do not plan to inform the result to the study participants unless they apply for it.

For peer review only

ETHICS AND DISSEMINATION

Ethical approval has been granted by the North of Scotland Research and Ethics Committee, reference: 20/NS/0105, 1 October 2020. This manuscript reflects the latest protocol V.7 approved 29 October 2020.

The study will be conducted according to the principles of Good Clinical Practice, General data Protection Regulation and Data Protection Act 2018 for Health and care Research. The sponsor and study team will ensure approval of the study protocol, participant information sheets, consent forms, letters to general practitioners and supporting documents by the appropriate regulatory body and research and ethics committee prior to participant recruitment. Documents will be stored securely with restricted access for at least 10 years. Written, informed consent will be obtained from each patient and an identification number provided. Any published data will not contain personal identifiable data. The results of this study will be disseminated by presentation at academic national/international conferences and publication in peer-review journals.

For peer review only

Author affiliations

[1] National Institute for Health Research Southampton Biomedical Research Centre, University of Southampton and University Hospital Southampton NHS Foundation Trust, Southampton, UK.

[2] School of Human Development and Health, Faculty of Medicine, University of Southampton, Southampton, UK.

[3] University Hospital Southampton NHS Foundation Trust, Southampton, UK.

[4] Southampton Clinical Trials Unit, University Hospital Southampton NHS Foundation Trust, Southampton, UK.

For peer review only

Acknowledgements: The authors will like to thank all the patients and healthy subjects who participated in this study.

Authors' contributions: ZH and PA have designed the study concept and formulated the initial idea; ZH is the Chief investigator and responsible for protocol review. DM and PA wrote the initial protocol. CB, PA, SW and ZH were involved in statistical analysis plan. DM and ZH was responsible for organising the study operation requirements, identifying and discussion with eligible participants. DM is responsible for patient recruitment and consent. DM and PA prepared the test meal and obtained the samples from the participants, VG is sorting the standard operating procedures (SOPs) and logistics with biobanking and sample process. PA is responsible for the analysis of the breath samples and the interpretation of the results from the breath test. JW is manging the study team and logistics related to clinical trial involvement. PA, DM, CB, SW, VG, JW and ZH were involved in drafting of the protocol.

Funding: This work is supported by Cancer Research UK (grant number C45617/A29908). DM partly funded by NIHR-BRC fellowship grant number NIHR-INF-0953.

Competing interest: None.

Patient consent for publication: Not required.

Provenance and peer review: Not commissioned; externally peer reviewed.

ORCID IDs

Paul R Afolabi https://orcid.org/0000-0002-0553-1578

Zaed Z Hamady https://orcid.org/0000-0002-4591-5226

References:

1. (UK) NGA. Pancreatic cancer in adults: diagnosis and management 2018. https://www.nice.org.uk/guidance/ng85.
2. Roberts KJ, Prasad P, Steele Y, et al. A reduced time to surgery within a 'fast track' pathway for periampullary malignancy is associated with an increased rate of pancreatoduodenectomy. *HPB (Oxford)* 2017;19(8):713-20. doi: 10.1016/j.hpb.2017.04.011 [published Online First: 2017/06/02]
3. Keane MG, Horsfall L, Rait G, et al. A case-control study comparing the incidence of early symptoms in pancreatic and biliary tract cancer. *Bmj Open* 2014;4(11) doi: ARTN e005720
10.1136/bmjopen-2014-005720
4. Lyratzopoulos G, Abel GA, McPhail S, et al. Measures of promptness of cancer diagnosis in primary care: secondary analysis of national audit data on patients with 18 common and rarer cancers. *Br J Cancer* 2013;108(3):686-90. doi: 10.1038/bjc.2013.1 [published Online First: 2013/02/09]
5. Lyratzopoulos G, Neal RD, Barbiere JM, et al. Variation in number of general practitioner consultations before hospital referral for cancer: findings from the 2010 National Cancer Patient Experience Survey in England. *Lancet Oncol* 2012;13(4):353-65. doi: 10.1016/S1470-2045(12)70041-4 [published Online First: 2012/03/01]
6. Chen W, Butler RK, Lustigova E, et al. Validation of the Enriching New-Onset Diabetes for Pancreatic Cancer Model in a Diverse and Integrated Healthcare Setting. *Dig Dis Sci* 2021;66(1):78-87. doi: 10.1007/s10620-020-06139-z [published Online First: 2020/03/01]
7. Chari ST, Leibson CL, Rabe KG, et al. Pancreatic cancer-associated diabetes mellitus: prevalence and temporal association with diagnosis of cancer. *Gastroenterology* 2008;134(1):95-101. doi: 10.1053/j.gastro.2007.10.040 [published Online First: 2007/12/07]
8. Action. PC. GPs dub it the 'silent killer' yet just 3% say they are fully confident in recognising symptoms. 2016 [Available from: pancreaticcanceraction.org/news/gps-dub-it-the-silent-killer-yet-just-3-say-they-are-fully-confident-in-recognising-symptoms/.
9. Collins GS, Altman DG. Identifying patients with undetected pancreatic cancer in primary care: an independent and external validation of QCancer((R)) (Pancreas). *Br J Gen Pract* 2013;63(614):e636-42. doi: 10.3399/bjgp13X671623 [published Online First: 2013/09/04]
10. Bartel MJ, Asbun H, Stauffer J, et al. Pancreatic exocrine insufficiency in pancreatic cancer: A review of the literature. *Dig Liver Dis* 2015;47(12):1013-20. doi: 10.1016/j.dld.2015.06.015 [published Online First: 2015/07/28]
11. Vujasinovic M, Valente R, Del Chiaro M, et al. Pancreatic Exocrine Insufficiency in Pancreatic Cancer. *Nutrients* 2017;9(3) doi: 10.3390/nu9030183 [published Online First: 2017/03/01]
12. Perez MM, Newcomer AD, Moertel CG, et al. Assessment of weight loss, food intake, fat metabolism, malabsorption, and treatment of pancreatic insufficiency in pancreatic cancer. *Cancer* 1983;52(2):346-52. doi: 10.1002/1097-0142(19830715)52:2<346::aid-cncr2820520228>3.0.co;2-z [published Online First: 1983/07/15]

For peer review only

13. Kato H, Nakao A, Kishimoto W, et al. 13C-labeled trioctanoin breath test for exocrine pancreatic function test in patients after pancreatoduodenectomy. *Am J Gastroenterol* 1993;88(1):64-9. [published Online First: 1993/01/01]
14. Matsumoto J, Traverso LW. Exocrine function following the whipple operation as assessed by stool elastase. *J Gastrointest Surg* 2006;10(9):1225-9. doi: 10.1016/j.gassur.2006.08.001 [published Online First: 2006/11/23]
15. Sikkens EC, Cahen DL, de Wit J, et al. Prospective assessment of the influence of pancreatic cancer resection on exocrine pancreatic function. *Br J Surg* 2014;101(2):109-13. doi: 10.1002/bjs.9342 [published Online First: 2013/12/18]
16. Sikkens EC, Cahen DL, de Wit J, et al. A prospective assessment of the natural course of the exocrine pancreatic function in patients with a pancreatic head tumor. *J Clin Gastroenterol* 2014;48(5):e43-6. doi: 10.1097/MCG.0b013e31829f56e7 [published Online First: 2014/04/11]
17. Levy P, Barthet M, Mollard BR, et al. Estimation of the prevalence and incidence of chronic pancreatitis and its complications. *Gastroenterol Clin Biol* 2006;30(6-7):838-44. doi: 10.1016/s0399-8320(06)73330-9 [published Online First: 2006/08/04]
18. Van de Vijver E, Desager K, Mulberg AE, et al. Treatment of infants and toddlers with cystic fibrosis-related pancreatic insufficiency and fat malabsorption with pancrelipase MT. *J Pediatr Gastroenterol Nutr* 2011;53(1):61-4. doi: 10.1097/MPG.0b013e31820e208e [published Online First: 2011/06/23]
19. Gonzalez-Sanchez V, Amrani R, Gonzalez V, et al. Diagnosis of exocrine pancreatic insufficiency in chronic pancreatitis: (13)C-Mixed Triglyceride Breath Test versus Fecal Elastase. *Pancreatology* 2017;17(4):580-85. doi: 10.1016/j.pan.2017.03.002 [published Online First: 2017/03/16]
20. Dominguez-Munoz JE, Iglesias-Garcia J, Vilarino-Insua M, et al. 13C-mixed triglyceride breath test to assess oral enzyme substitution therapy in patients with chronic pancreatitis. *Clin Gastroenterol Hepatol* 2007;5(4):484-8. doi: 10.1016/j.cgh.2007.01.004 [published Online First: 2007/04/21]
21. Muniz CK, dos Santos JS, Pfrimer K, et al. Nutritional status, fecal elastase-1, and 13C-labeled mixed triglyceride breath test in the long-term after pancreaticoduodenectomy. *Pancreas* 2014;43(3):445-50. doi: 10.1097/MPA.0000000000000048 [published Online First: 2014/03/14]

For peer review only

Figure 1 Proposed diagnostic tool consisting of faecal elastase-1 test and the ^{13}C-mixed triglyceride breath test to screen for pancreatic exocrine dysfunction in patients ‘at-risk’ of pancreatic cancer.

For peer review only

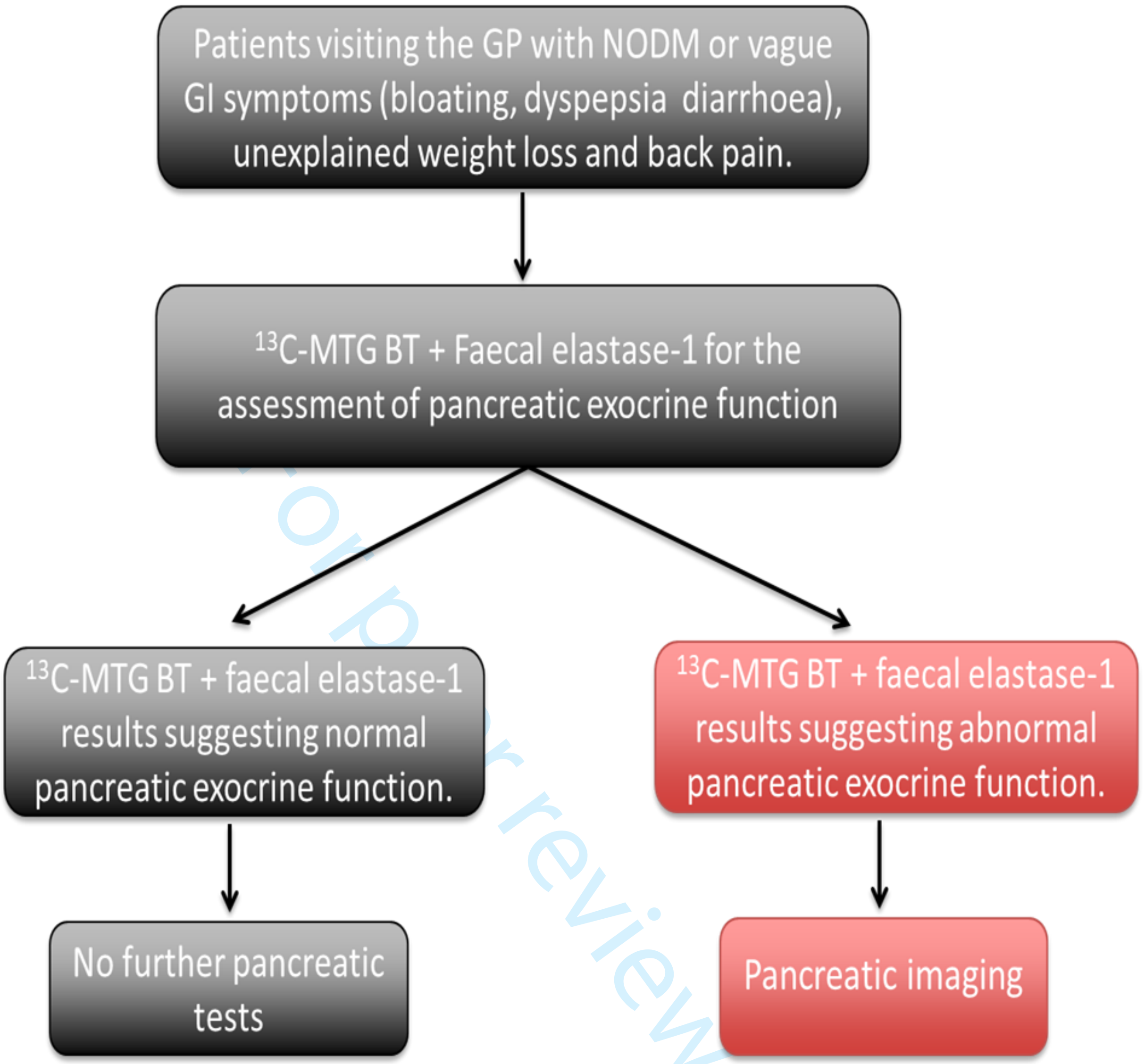

Abbreviations: NODM- New Onset Diabetes Mellitus, ^{13}C-MTG BT- ^{13}C-mixed triglyceride breath test.

For peer review only

BMJ Open

DEPEND study protocol: Early detection of patients with pancreatic cancer: a pilot study to evaluate the utility of faecal elastase-1 and ^{13}C-mixed triglyceride breath test as screening tools in high-risk individuals.

Journal:	*BMJ Open*
Manuscript ID	bmjopen-2021-057271.R1
Article Type:	Protocol
Date Submitted by the Author:	12-Dec-2021
Complete List of Authors:	Afolabi, Paul; University of Southampton Faculty of Medicine; NIHR Southampton Biomedical Research Centre, Southampton Centre for Biomedical Research Mass Spectrometry<br>McDonnell, Declan; University of Southampton Faculty of Medicine; NIHR Southampton Biomedical Research Centre, Southampton Centre for Biomedical Research<br>Byrne, C. D.; University of Southampton Faculty of Medicine; NIHR Southampton Biomedical Research Centre, Southampton Centre for Biomedical Research<br>Wilding, Sam; University Hospital Southampton NHS Foundation Trust, Southampton Clinical Trials Unit.<br>Goss, Victoria; University Hospital Southampton NHS Foundation Trust, Southampton Clinical Trials Unit<br>Walters, Jocelyn; University Hospital Southampton NHS Foundation Trust, Southampton Clinical Trials Unit.<br>Hamady, Zaed; University Hospital Southampton NHS Foundation Trust; NIHR Southampton Biomedical Research Centre, Southampton Centre for Biomedical Research
**Primary Subject Heading**:	Oncology
Secondary Subject Heading:	Diagnostics, Gastroenterology and hepatology, Oncology
Keywords:	Endocrine tumours < DIABETES & ENDOCRINOLOGY, Gastrointestinal tumours < GASTROENTEROLOGY, Pancreatic surgery < SURGERY, Biochemistry < NATURAL SCIENCE DISCIPLINES, Pancreatic disease < GASTROENTEROLOGY

SCHOLARONE™
Manuscripts

DEPEND study protocol: Early detection of patients with pancreatic cancer: a pilot study to evaluate the utility of faecal elastase-1 and ^{13}C-mixed triglyceride breath test as screening tools in high-risk individuals.

Paul R Afolabi [1,2], Declan McDonnell [1,2,3], Christopher D Byrne [1,2,3] , Sam Wilding [4], Victoria M Goss [4], Jocelyn Walters [4], Zaed Z Hamady [1,2,3].

Correspondence to Mr Zaed Hamady; Z.Hamady@soton.ac.uk

For peer review only - http://bmjopen.bmj.com/site/about/guidelines.xhtml

Abstract

Introduction Pancreatic cancer (PC) is the fifth leading cause of cancer related death in the UK. The incidence of PC is increasing, with little or no improvement in overall survival and the best chance for long-term survival in patients with PC relies on early detection and surgical resection. In this study, we propose the use of a diagnostic algorithm that combines tests of pancreatic exocrine function (Faecal elastase-1 test (FE-1) and the ^{13}C-mixed triglyceride (^{13}C-MTG) breath test) to identify patients with PC that urgently needs imaging studies.

Methods and analysis This prospective pilot (proof of concept) study will be carried out on 25 patients with resectable PC, 10 patients with chronic pancreatitis and 25 healthy volunteers. This study will construct a predictive algorithm for PC, utilising two tests of pancreatic exocrine function, FE-1 test and the ^{13}C-MTG breath test. Continuous flow isotope ratio mass spectrometry in the ^{13}C-MTG breath test will be used to analyse enriched $^{13}CO_2$ in exhaled breath samples. The additional predictive benefit of other potential biomarkers of PC will also be considered. Potential biomarkers of PC showing abilities to discriminate between patients with PC from healthy volunteers or patients with chronic pancreatitis will be selected by metabolomic analysis.

Ethics and dissemination The study was approved by the North of Scotland Research and Ethics Committee on 1 October 2020 (reference: 20/NS/0105, favourable opinion granted). The results will be disseminated in presentations at academic national/international conferences and publication in peer-review journals.

For peer review only - http://bmjopen.bmj.com/site/about/guidelines.xhtml

Strength and limitations of this study

- This pilot study is aiming to be the first study to construct a predictive algorithm for the early detection of pancreatic cancer (PC) based on tests of pancreatic exocrine function.
- PC patients with potentially curative pancreatic resection will be recruited in this study.
- In this study, we would expect chronic pancreatitis to also have positive findings using the exocrine function tests. However, we are not proposing to use the faecal elastase-1 test and ^{13}C-mixed triglyceride breath test to differentiate between chronic pancreatitis and pancreatic cancer at this stage.
- Participants' return of faecal specimens may be limited by embarrassment, hesitancy or inability of the study participants to provide a sample.

For peer review only

For peer review only - http://bmjopen.bmj.com/site/about/guidelines.xhtml

INTRODUCTION

Pancreatic cancer (PC) is the fifth leading cause of cancer related death in the UK. The UK has one of the worst survival rates in Europe, with an average life expectancy at diagnosis of just 4–6 months and 20% survival at 1 year. In addition, in the UK, only about 7% of people survive for 5 years or longer[1]**.** Since complete surgical resection remains the only potentially curative option for PC, it is crucial to identify PC at an early stage, when it is still resectable. Unfortunately, only 10–20% of patients with PC are diagnosed at a stage when curative surgery remains an option[2].

The early symptoms of PC are usually vague including weight loss, upper abdominal pain, backache, fatigue, diarrhoea and nausea. In 93% of patients, these symptoms may have been present for up to two years before diagnosis of PC [3]. Recent studies show that around 40% of patients diagnosed with PC require three or more visits to their GP before they are referred to a specialist [4 5] thereby leading to a delay in the diagnosis. There is also an increased risk of developing PC in those with new-onset diabetes mellitus (NODM) i.e. less than three years since diagnosis [6] and as many as 25% of PC cases are believed to present with NODM [7].

Recent survey of GPs commissioned by Pancreatic Cancer Action [8] suggested that the delay between not having access to a reliable diagnostic referral pathway and a confirmed diagnosis of PC is a major barrier to the early detection of PC. As the symptoms of PC are often non-specific and unlikely to prompt further clinical investigation, there is an urgent need to have a reliable and cheap screening test that can be used to identify high-risk individuals who require urgent imaging studies such as CT scan to confirm or exclude the

For peer review only - http://bmjopen.bmj.com/site/about/guidelines.xhtml

presence of a pancreatic mass [9]. Having such a test with reliable sensitivity and negative predictive value (NPV), would enable the GP to select patients for urgent referral to further investigations of PC. A high NPV value would enable the GP to reassure the patient that they did not need further imaging studies. Our innovative approach is to use tests of pancreatic exocrine function combined as a screening test in patients presenting with the vague symptoms that may be associated with PC.

Although pancreatic exocrine insufficiency (PEI) is a well-known complication of pancreatic cancer [10 11], its presence is frequently overlooked in patients with advanced PC resulting in a decreased quality of life (QoL), malnutrition and morbidity [12]. Currently, the diagnostic tests of pancreatic exocrine function such as faecal elastase-1 (FE-1) or the ^{13}C-mixed triglyceride (^{13}C-MTG) breath test, may be used to detect PEI in patients after pancreatic surgery and chronic pancreatitis [13-16].

FE-1 test is a simple indirect and non-invasive method for assessing pancreatic secretion, which measures faecal concentrations of elastase-1, a proteolytic enzyme produced exclusively by pancreatic acinar cells, which binds to bile salts and passes through the gut with minimal degradation. FE-1 level correlate well with output of other pancreatic enzymes, such as amylase, lipase and trypsin [17 18] and have been shown to detect PEI in patients with advanced PC and following pancreatic surgery [14-16].

The ^{13}C-MTG breath test assesses pancreatic lipase, it involves the ingestion of a ^{13}C-labeled mixed triglyceride (1,3-distearoyl, 2- (carboxyl-^{13}C)-octanoyl glycerol) mixed with a test meal. The ^{13}C-MTG contains a ^{13}C-labeled medium-chain fatty acid (^{13}C-octanoate) at the Sn-

For peer review only - http://bmjopen.bmj.com/site/about/guidelines.xhtml

2 position, and two long-chain fatty acids (stearic acid) at the Sn-1 and Sn-3 positions of the glycerol backbone of the triacylglycerol. The ingested ^{13}C-MTG then undergoes the lipolysis of the two-long chain fatty acids from ^{13}C-MTG by pancreatic lipase to produce free fatty acids (stearic acid and ^{13}C-octanoate) and monoacylglycerol. After the intestinal absorption, the ^{13}C-free fatty acids are oxidised in the liver producing $^{13}CO_2$, which is then released on breath. Therefore, the $^{13}CO_2$ concentration in breath reflects pancreatic lipase activity. The ^{13}C-MTG breath test can be an alternative or complementary to the FE-1 test for PEI. The ^{13}C-MTG breath test has been validated in patients with chronic pancreatitis [19] and PC following surgical resection of the cancer [20 21], but not prior to surgery in patients with resectable PC.

Both FE-1 and the ^{13}C-MTG breath test are indirect tests of pancreatic exocrine function. To date, only one study has shown the performance of the ^{13}C-trioctanoin breath test with a sensitivity of 100% to detect the presence of PC in 14 patients [13] while another study has shown that a FE-1 value of ⩽ 20μg/g is an independent predictor of poor survival in patients with advanced PC [22].

In this study, we will validate FE-1 test and ^{13}C-MTG breath test as potential diagnostic biomarkers of resectable PC and test their performance in discriminating between patients with PC and healthy subjects. We aim to assess the diagnostic performances of the exocrine function tests (as a combined diagnostic panel) in detecting PC. If so, then a logistic regression analysis will be performed that will integrate the results of FE-1 test and the ^{13}C-MTG breath test along with other potential biomarkers of PC as a diagnostic algorithm panel.

For peer review only - http://bmjopen.bmj.com/site/about/guidelines.xhtml

These tests can provide a non-invasive risk stratification tool to identify patients at risk of having PC when they present with NODM, weight loss or vague upper GI symptoms. Furthermore, this will provide a rational approach to patients identified to have PEI to be selected to have pancreatic imaging to confirm the presence of PC and having a pancreatic resection earlier (Figure 1). In addition, we will be identifying other potential biomarkers of PC through unbiased multi-omics analysis of blood samples (proteomics and metabolomics).

For peer review only

For peer review only - http://bmjopen.bmj.com/site/about/guidelines.xhtml

METHODS AND ANALYSIS

Main centre

NIHR Southampton Biomedical Research Centre, Southampton Centre for Biomedical Research, Southampton General Hospital, Southampton, UK.

Dates of the study

From 16th March 2021 to 16th March 2022.

Design

This is a single cross-sectional pilot study of patients with PC, chronic pancreatitis and healthy controls, matched for age and sex. The primary objective is to determine if ^{13}C-mixed triglyceride (^{13}C-MTG) breath test and faecal elastase-1 (FE-1) concentrations combined can discriminate between the groups. Patients with PC will be recruited at the pre-operative stage within two weeks of initial diagnosis. The secondary objective of this study is the analysis of multi-omic (proteomic and metabolomic) profiles and comparison between the groups.

Inclusion criteria

Male and female participants aged from 30-85 within the Wessex pancreatic cancer catchment area will be suitable for inclusion. In the cancer cohort, patients with a diagnosis of resectable pancreatic ductal adenocarcinoma (PDAC) (stage 1-2) will be included. Patients with chronic pancreatitis and healthy subjects will also be recruited as control groups and matched for age and sex to the cases of PDAC.

Exclusion criteria

Patients and controls with autoimmune or other chronic inflammatory conditions, or those who have previously had pancreatic disease or liver dysfunction will be excluded.

For peer review only

For peer review only - http://bmjopen.bmj.com/site/about/guidelines.xhtml

Intervention(s) or method

Each participant will be given a participant information sheet explaining the research and will sign an informed consent form. Participants are required to fast overnight for at least 12 hours prior to undergoing the ^{13}C-mixed triglyceride (^{13}C-MTG). At the Clinical Research Facility (CRF), full infection control precautions (related to COVID-19 pandemic) will be adhered to throughout the visit. Participants will have their temperature checked on arrival to ensure they are not pyrexial as part of screening for Covid-19, which will exclude them from the study. Anthropometric measurements (height, weight and waist circumference) of the participants will be recorded. Baseline blood samples will be taken prior to undergoing the ^{13}C-MTG breath test. The data collected will include the following: Full blood count (FBC), Urea and Electrolyte blood test (U&E), Liver function tests (AST, ALT, ALP, albumin and GGT) /clotting studies, glycated hemoglobulin (HbA1c %), Lipid profile, Vitamins and micronutrients, Hyaluronic acid, Procollagen III amino terminal peptide (PIINP) and Tissue inhibitor of metalloproteinase-1 (TIMP-1). These tests will be analysed immediately after the blood has been drawn from the participants by the chemical pathology service laboratory at Southampton General Hospital. Further blood samples will be processed and stored for multi-omics analysis. Participants will also be asked to provide spot stool samples for faecal elastase-1 (FE-1) concentrations by using a commercially available ELISA kit. Results of the FE-1 test will be expressed as µg/g of stool.

For peer review only

For peer review only - http://bmjopen.bmj.com/site/about/guidelines.xhtml

^{13}C-MTG breath test protocol

Each participant will be asked to provide 2 baseline breath samples by blowing into 2 × 10ml exetainer tubes through a straw while wearing a facemask. After that, participants will be given an oral dose of 250mg of 2-[^{13}C]-octanoyl-1,3-distearin (^{13}C-MTG -Cambridge Isotope Laboratories, Andover, MA, USA) together with a solid test meal consisting of a crispbread or other gluten-free alternative, and 200ml of water. Post-prandial breath samples will be then collected every 30mins for 4 hours. No food or drinks except for water will be allowed during the duration of the breath test study. The collected breath samples will be analysed for the ratio of $^{13}CO_2$ to $^{12}CO_2$ using a Continuous Flow Isotope Ratio Mass Spectrometer (CF-IRMS SERCON Ltd, Crewe, UK) at the Southampton Centre for Biomedical Research. The increase in $^{13}CO_2$ content with regard to the baseline value of the initial breath sample is expressed as atom % excess and we will express the results of the ^{13}C-MTG breath test as the cumulative percentage of ^{13}C-label recovered on breath as $^{13}CO_2$ over 4 hours (cPDR over 4 hours).

Primary Outcome: Prediction of PC using a logistic model with measurement of FE-1 concentration expressed as µg/g of stool and ^{13}C-MTG breath test expressed as cPDR over 4hrs as covariates.

For peer review only - http://bmjopen.bmj.com/site/about/guidelines.xhtml

Statistical analysis and data analysis plans

Sample size calculation

The sample size calculation for the proposed study is based on a previous study assessing pancreatic exocrine function using a ^{13}C-triglyceride breath test in healthy subjects and patients with a localised pancreatic mass [13]. In this study, they used the ^{13}C-trioctanoin breath test to assess pancreatic exocrine function in 14 patients with a localised pancreatic mass and 5 healthy control subjects. The results of the study showed means ± standard deviation (SD) of the recovery of $^{13}CO_2$ over 3hrs after undergoing the ^{13}C-trioctanoin breath test of 42% ± 3.4% for the healthy controls and 24.2% ± 10.5% for patients with a localised pancreatic mass with an effect size or mean difference between both groups of 17.8% and a within group SD of 9.1% (based on the SD estimates of 3.4% and 10.5% for each group respectively).

Using data from this study, we calculated that a sample size of 25 subjects in each group (patients with PC, patients with chronic pancreatitis and healthy control subjects) will give 100% power to detect a difference of 17.8% between patients with PC and healthy control group assuming a standard deviation of 10.5% in patients with PC at a significance level of 5% using a two-tailed test. The power calculation was carried out using IBM SPSS SamplePower version 3.

For peer review only - http://bmjopen.bmj.com/site/about/guidelines.xhtml

Statistical tests

Data will be presented as mean + SD, median + IQR when appropriate and 95% CI. Comparison of results of FE-1 and the ^{13}C-MTG breath test between each group will be evaluated by employing either the one way analysis of variance test (if the variables are normally distributed and parametric validity conditions are fulfilled) or the Mann Whitney U-test or Kruskal-Wallis test (if the variables are not normally distributed and non-parametric validity conditions are fulfilled). A multi-variable logistic regression analysis will be performed in order to determine the independent factors that are significantly associated with the presence of PDAC and logistic regression models will be constructed based on the identified independent factors. Furthermore, multi-variable regression analysis will be used to determine the optimal algorithm that combines the results of FE-1 test and ^{13}C-MTG breath test. We will also test whether potential biomarkers of PC identified by unbiased omics analysis improve the prediction of PDAC. A Receiver operating characteristic (ROC) curve will be constructed to evaluate the diagnostic accuracy (sensitivity, specificity, positive and negative predictive value, receiver operator curve analysis) of the algorithm for PDAC. Furthermore, the optimal cut off point for the algorithm to identify patients with PC will be estimated using the Youden index. A two-sided p value of <0.05 will be considered to indicate statistical significance. All analysis will be performed using SPSS (IBM, V.25.0) or Stata (Statacorp, v16.0).

Patient and public involvement statement

Patients, healthy subjects and the public were not involved in the study design, recruitment and conduct. We do not plan to inform the result to the study participants unless they apply for it.

For peer review only - http://bmjopen.bmj.com/site/about/guidelines.xhtml

ETHICS AND DISSEMINATION

Ethical approval has been granted by the North of Scotland Research and Ethics Committee, reference: 20/NS/0105, 1 October 2020. This manuscript reflects the latest protocol V.7 approved 29 October 2020.

The study will be conducted according to the principles of Good Clinical Practice, General data Protection Regulation and Data Protection Act 2018 for Health and care Research. The sponsor and study team will ensure approval of the study protocol, participant information sheets, consent forms, letters to general practitioners and supporting documents by the appropriate regulatory body and research and ethics committee prior to participant recruitment. Documents will be stored securely with restricted access for at least 10 years. Written, informed consent will be obtained from each patient and an identification number provided. Any published data will not contain personal identifiable data. The results of this study will be disseminated by presentation at academic national/international conferences and publication in peer-review journals.

For peer review only

For peer review only - http://bmjopen.bmj.com/site/about/guidelines.xhtml

Author affiliations

[1] National Institute for Health Research Southampton Biomedical Research Centre, University of Southampton and University Hospital Southampton NHS Foundation Trust, Southampton, UK.

[2] School of Human Development and Health, Faculty of Medicine, University of Southampton, Southampton, UK.

[3] University Hospital Southampton NHS Foundation Trust, Southampton, UK.

[4] Southampton Clinical Trials Unit, University Hospital Southampton NHS Foundation Trust, Southampton, UK.

Acknowledgements: The authors will like to thank all the patients and healthy subjects who participated in this study.

Authors' contributions: ZH and PA have designed the study concept and formulated the initial idea; ZH is the Chief investigator and responsible for protocol review. DM and PA wrote the initial protocol. CB, PA, SW and ZH were involved in statistical analysis plan. DM and ZH was responsible for organising the study operation requirements, identifying and discussion with eligible participants. DM is responsible for patient recruitment and consent. DM and PA prepared the test meal and obtained the samples from the participants, VG is sorting the standard operating procedures (SOPs) and logistics with biobanking and sample process. PA is responsible for the analysis of the breath samples and the interpretation of the results from the breath test. JW is manging the study team and logistics related to clinical trial involvement. PA, DM, CB, SW, VG, JW and ZH were involved in drafting of the protocol.

Funding: This work is supported by Cancer Research UK (grant number C45617/A29908). DM partly funded by NIHR-BRC fellowship grant number NIHR-INF-0953.

Competing interest: None.

Patient consent for publication: Not required.

Provenance and peer review: Not commissioned; externally peer reviewed.

ORCID IDs

Paul R Afolabi https://orcid.org/0000-0002-0553-1578

Zaed Z Hamady https://orcid.org/0000-0002-4591-5226

For peer review only

References:

1. Allemani C, Matsuda T, Di Carlo V, et al. Global surveillance of trends in cancer survival 2000–14 (CONCORD-3): analysis of individual records for 37 513 025 patients diagnosed with one of 18 cancers from 322 population-based registries in 71 countries. *The Lancet* 2018;391(10125):1023-75. doi: 10.1016/s0140-6736(17)33326-3
2. Roberts KJ, Prasad P, Steele Y, et al. A reduced time to surgery within a 'fast track' pathway for periampullary malignancy is associated with an increased rate of pancreatoduodenectomy. *HPB (Oxford)* 2017;19(8):713-20. doi: 10.1016/j.hpb.2017.04.011 [published Online First: 2017/06/02]
3. Keane MG, Horsfall L, Rait G, et al. A case-control study comparing the incidence of early symptoms in pancreatic and biliary tract cancer. *Bmj Open* 2014;4(11) doi: ARTN e005720

10.1136/bmjopen-2014-005720

4. Lyratzopoulos G, Abel GA, McPhail S, et al. Measures of promptness of cancer diagnosis in primary care: secondary analysis of national audit data on patients with 18 common and rarer cancers. *Br J Cancer* 2013;108(3):686-90. doi: 10.1038/bjc.2013.1 [published Online First: 2013/02/09]
5. Lyratzopoulos G, Neal RD, Barbiere JM, et al. Variation in number of general practitioner consultations before hospital referral for cancer: findings from the 2010 National Cancer Patient Experience Survey in England. *Lancet Oncol* 2012;13(4):353-65. doi: 10.1016/S1470-2045(12)70041-4 [published Online First: 2012/03/01]
6. Chen W, Butler RK, Lustigova E, et al. Validation of the Enriching New-Onset Diabetes for Pancreatic Cancer Model in a Diverse and Integrated Healthcare Setting. *Dig Dis Sci* 2021;66(1):78-87. doi: 10.1007/s10620-020-06139-z [published Online First: 2020/03/01]
7. Chari ST, Leibson CL, Rabe KG, et al. Pancreatic cancer-associated diabetes mellitus: prevalence and temporal association with diagnosis of cancer. *Gastroenterology* 2008;134(1):95-101. doi: 10.1053/j.gastro.2007.10.040 [published Online First: 2007/12/07]
8. Action. PC. GPs dub it the 'silent killer' yet just 3% say they are fully confident in recognising symptoms. 2016 [Available from: pancreaticcanceraction.org/news/gps-dub-it-the-silent-killer-yet-just-3-say-they-are-fully-confident-in-recognising-symptoms/.
9. Collins GS, Altman DG. Identifying patients with undetected pancreatic cancer in primary care: an independent and external validation of QCancer((R)) (Pancreas). *Br J Gen Pract* 2013;63(614):e636-42. doi: 10.3399/bjgp13X671623 [published Online First: 2013/09/04]
10. Bartel MJ, Asbun H, Stauffer J, et al. Pancreatic exocrine insufficiency in pancreatic cancer: A review of the literature. *Dig Liver Dis* 2015;47(12):1013-20. doi: 10.1016/j.dld.2015.06.015 [published Online First: 2015/07/28]
11. Vujasinovic M, Valente R, Del Chiaro M, et al. Pancreatic Exocrine Insufficiency in Pancreatic Cancer. *Nutrients* 2017;9(3) doi: 10.3390/nu9030183 [published Online First: 2017/03/01]
12. Perez MM, Newcomer AD, Moertel CG, et al. Assessment of weight loss, food intake, fat metabolism, malabsorption, and treatment of pancreatic insufficiency in pancreatic cancer. *Cancer* 1983;52(2):346-52. doi: 10.1002/1097-0142(19830715)52:2<346::aid-cncr2820520228>3.0.co;2-z [published Online First: 1983/07/15]

For peer review only - http://bmjopen.bmj.com/site/about/guidelines.xhtml

13. Kato H, Nakao A, Kishimoto W, et al. 13C-labeled trioctanoin breath test for exocrine pancreatic function test in patients after pancreatoduodenectomy. *Am J Gastroenterol* 1993;88(1):64-9. [published Online First: 1993/01/01]
14. Matsumoto J, Traverso LW. Exocrine function following the whipple operation as assessed by stool elastase. *J Gastrointest Surg* 2006;10(9):1225-9. doi: 10.1016/j.gassur.2006.08.001 [published Online First: 2006/11/23]
15. Sikkens EC, Cahen DL, de Wit J, et al. Prospective assessment of the influence of pancreatic cancer resection on exocrine pancreatic function. *Br J Surg* 2014;101(2):109-13. doi: 10.1002/bjs.9342 [published Online First: 2013/12/18]
16. Sikkens EC, Cahen DL, de Wit J, et al. A prospective assessment of the natural course of the exocrine pancreatic function in patients with a pancreatic head tumor. *J Clin Gastroenterol* 2014;48(5):e43-6. doi: 10.1097/MCG.0b013e31829f56e7 [published Online First: 2014/04/11]
17. Levy P, Barthet M, Mollard BR, et al. Estimation of the prevalence and incidence of chronic pancreatitis and its complications. *Gastroenterol Clin Biol* 2006;30(6-7):838-44. doi: 10.1016/s0399-8320(06)73330-9 [published Online First: 2006/08/04]
18. Van de Vijver E, Desager K, Mulberg AE, et al. Treatment of infants and toddlers with cystic fibrosis-related pancreatic insufficiency and fat malabsorption with pancrelipase MT. *J Pediatr Gastroenterol Nutr* 2011;53(1):61-4. doi: 10.1097/MPG.0b013e31820e208e [published Online First: 2011/06/23]
19. Gonzalez-Sanchez V, Amrani R, Gonzalez V, et al. Diagnosis of exocrine pancreatic insufficiency in chronic pancreatitis: (13)C-Mixed Triglyceride Breath Test versus Fecal Elastase. *Pancreatology* 2017;17(4):580-85. doi: 10.1016/j.pan.2017.03.002 [published Online First: 2017/03/16]
20. Dominguez-Munoz JE, Iglesias-Garcia J, Vilarino-Insua M, et al. 13C-mixed triglyceride breath test to assess oral enzyme substitution therapy in patients with chronic pancreatitis. *Clin Gastroenterol Hepatol* 2007;5(4):484-8. doi: 10.1016/j.cgh.2007.01.004 [published Online First: 2007/04/21]
21. Muniz CK, dos Santos JS, Pfrimer K, et al. Nutritional status, fecal elastase-1, and 13C-labeled mixed triglyceride breath test in the long-term after pancreaticoduodenectomy. *Pancreas* 2014;43(3):445-50. doi: 10.1097/MPA.0000000000000048 [published Online First: 2014/03/14]
22. Partelli S, Frulloni L, Minniti C, et al. Faecal elastase-1 is an independent predictor of survival in advanced pancreatic cancer. *Dig Liver Dis* 2012;44(11):945-51. doi: 10.1016/j.dld.2012.05.017 [published Online First: 2012/07/04]

For peer review only - http://bmjopen.bmj.com/site/about/guidelines.xhtml

Figure 1 Proposed diagnostic tool consisting of faecal elastase-1 test and the ^{13}C-mixed triglyceride breath test to screen for pancreatic exocrine dysfunction in patients 'at-risk' of pancreatic cancer.

For peer review only

For peer review only - http://bmjopen.bmj.com/site/about/guidelines.xhtml

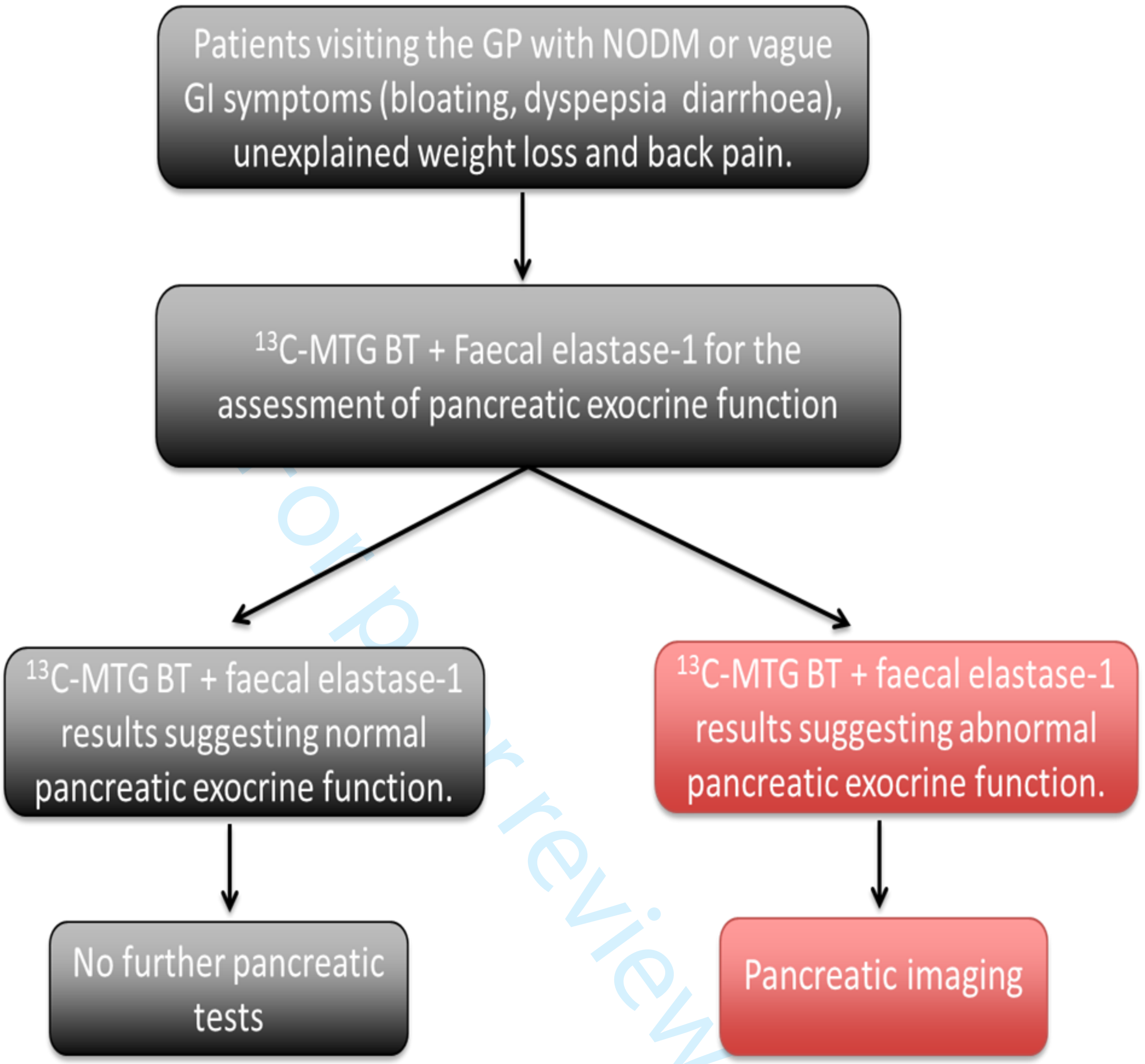

Abbreviations: NODM- New Onset Diabetes Mellitus, ^{13}C-MTG BT- ^{13}C-mixed triglyceride breath test.

TRIPOD Checklist: Prediction Model Development

Section/Topic	Item	Checklist Item	Page
**Title and abstract**			
Title	1	Identify the study as developing and/or validating a multivariable prediction model, the target population, and the outcome to be predicted.	Page 1, lines 11-14
Abstract	2	Provide a summary of objectives, study design, setting, participants, sample size, predictors, outcome, statistical analysis, results, and conclusions.	Page 2, lines 22-43.
**Introduction**			
Background and objectives	3a	Explain the medical context (including whether diagnostic or prognostic) and rationale for developing or validating the multivariable prediction model, including references to existing models.	Pages 4-6, lines 57-130.
	3b	Specify the objectives, including whether the study describes the development or validation of the model or both.	Page 6, lines 124-130.
**Methods**			
Source of data	4a	Describe the study design or source of data (e.g., randomized trial, cohort, or registry data), separately for the development and validation data sets, if applicable.	Page 8, lines 145-152
	4b	Specify the key study dates, including start of accrual; end of accrual; and, if applicable, end of follow-up.	Page 8, lines 142-143.
Participants	5a	Specify key elements of the study setting (e.g., primary care, secondary care, general population) including number and location of centres.	Page 8, lines 139-141.
	5b	Describe eligibility criteria for participants.	Page 8, lines 153-161.
	5c	Give details of treatments received, if relevant.	n/a
Outcome	6a	Clearly define the outcome that is predicted by the prediction model, including how and when assessed.	Page 10, lines 203-205.
	6b	Report any actions to blind assessment of the outcome to be predicted.	n/a
Predictors	7a	Clearly define all predictors used in developing or validating the multivariable prediction model, including how and when they were measured.	n/a
	7b	Report any actions to blind assessment of predictors for the outcome and other predictors.	n/a
Sample size	8	Explain how the study size was arrived at.	Page 11, lines 215-222.
Missing data	9	Describe how missing data were handled (e.g., complete-case analysis, single imputation, multiple imputation) with details of any imputation method.	n/a
Statistical analysis methods	10a	Describe how predictors were handled in the analyses.	n/a
	10b	Specify type of model, all model-building procedures (including any predictor selection), and method for internal validation.	Page 12, lines 231-249.
	10d	Specify all measures used to assess model performance and, if relevant, to compare multiple models.	n/a
Risk groups	11	Provide details on how risk groups were created, if done.	n/a
**Results**			
Participants	13a	Describe the flow of participants through the study, including the number of participants with and without the outcome and, if applicable, a summary of the follow-up time. A diagram may be helpful.	n/a
	13b	Describe the characteristics of the participants (basic demographics, clinical features, available predictors), including the number of participants with missing data for predictors and outcome.	n/a
Model development	14a	Specify the number of participants and outcome events in each analysis.	n/a
	14b	If done, report the unadjusted association between each candidate predictor and outcome.	n/a
Model specification	15a	Present the full prediction model to allow predictions for individuals (i.e., all regression coefficients, and model intercept or baseline survival at a given time point).	n/a
	15b	Explain how to the use the prediction model.	n/a
Model performance	16	Report performance measures (with CIs) for the prediction model.	n/a
**Discussion**			
Limitations	18	Discuss any limitations of the study (such as nonrepresentative sample, few events per predictor, missing data).	n/a

TRIPOD Checklist: Prediction Model Development

Interpretation	19b	Give an overall interpretation of the results, considering objectives, limitations, and results from similar studies, and other relevant evidence.	n/a
Implications	20	Discuss the potential clinical use of the model and implications for future research.	n/a
**Other information**			
Supplementary information	21	Provide information about the availability of supplementary resources, such as study protocol, Web calculator, and data sets.	n/a
Funding	22	Give the source of funding and the role of the funders for the present study.	Page 14, lines 303-304.

We recommend using the TRIPOD Checklist in conjunction with the TRIPOD Explanation and Elaboration document.

For peer review only

BMJ Open

DEPEND study protocol: Early detection of patients with pancreatic cancer: a pilot study to evaluate the utility of faecal elastase-1 and ^{13}C-mixed triglyceride breath test as screening tools in high-risk individuals.

Journal:	*BMJ Open*
Manuscript ID	bmjopen-2021-057271.R2
Article Type:	Protocol
Date Submitted by the Author:	03-Feb-2022
Complete List of Authors:	Afolabi, Paul; University of Southampton Faculty of Medicine; NIHR Southampton Biomedical Research Centre, Southampton Centre for Biomedical Research Mass Spectrometry<br>McDonnell, Declan; University of Southampton Faculty of Medicine; NIHR Southampton Biomedical Research Centre, Southampton Centre for Biomedical Research<br>Byrne, C. D.; University of Southampton Faculty of Medicine; NIHR Southampton Biomedical Research Centre, Southampton Centre for Biomedical Research<br>Wilding, Sam; University Hospital Southampton NHS Foundation Trust, Southampton Clinical Trials Unit.<br>Goss, Victoria; University Hospital Southampton NHS Foundation Trust, Southampton Clinical Trials Unit<br>Walters, Jocelyn; University Hospital Southampton NHS Foundation Trust, Southampton Clinical Trials Unit.<br>Hamady, Zaed; University Hospital Southampton NHS Foundation Trust; NIHR Southampton Biomedical Research Centre, Southampton Centre for Biomedical Research
**Primary Subject Heading**:	Oncology
Secondary Subject Heading:	Diagnostics, Gastroenterology and hepatology, Oncology
Keywords:	Endocrine tumours < DIABETES & ENDOCRINOLOGY, Gastrointestinal tumours < GASTROENTEROLOGY, Pancreatic surgery < SURGERY, Biochemistry < NATURAL SCIENCE DISCIPLINES, Pancreatic disease < GASTROENTEROLOGY

SCHOLARONE™
Manuscripts

DEPEND study protocol: Early detection of patients with pancreatic cancer: a pilot study to evaluate the utility of faecal elastase-1 and ^{13}C-mixed triglyceride breath test as screening tools in high-risk individuals.

Paul R Afolabi [1,2], Declan McDonnell [1,2,3], Christopher D Byrne [1,2,3] , Sam Wilding [4], Victoria M Goss [4], Jocelyn Walters [4], Zaed Z Hamady [1,2,3].

Correspondence to Mr Zaed Hamady; Z.Hamady@soton.ac.uk

Author affiliations

[1] National Institute for Health Research Southampton Biomedical Research Centre, University of Southampton and University Hospital Southampton NHS Foundation Trust, Southampton, UK.

[2] School of Human Development and Health, Faculty of Medicine, University of Southampton, Southampton, UK.

[3] University Hospital Southampton NHS Foundation Trust, Southampton, UK.

[4] Southampton Clinical Trials Unit, University Hospital Southampton NHS Foundation Trust, Southampton, UK.

For peer review only - http://bmjopen.bmj.com/site/about/guidelines.xhtml

For peer review only

Abstract

Introduction Pancreatic cancer (PC) is the fifth leading cause of cancer related death in the UK. The incidence of PC is increasing, with little or no improvement in overall survival and the best chance for long-term survival in patients with PC relies on early detection and surgical resection. In this study, we propose the use of a diagnostic algorithm that combines tests of pancreatic exocrine function (Faecal elastase-1 test (FE-1) and the ^{13}C-mixed triglyceride (^{13}C-MTG) breath test) to identify patients with PC that urgently needs imaging studies.

Methods and analysis This prospective pilot (proof of concept) study will be carried out on 25 patients with resectable PC, 10 patients with chronic pancreatitis and 25 healthy volunteers. This study will construct a predictive algorithm for PC, utilising two tests of pancreatic exocrine function, FE-1 test and the ^{13}C-MTG breath test. Continuous flow isotope ratio mass spectrometry in the ^{13}C-MTG breath test will be used to analyse enriched $^{13}CO_2$ in exhaled breath samples. The additional predictive benefit of other potential biomarkers of PC will also be considered. Potential biomarkers of PC showing abilities to discriminate between patients with PC from healthy subjects or patients with chronic pancreatitis will be selected by metabolomic analysis.

Ethics and dissemination The study was approved by the North of Scotland Research and Ethics Committee on 1 October 2020 (reference: 20/NS/0105, favourable opinion granted). The results will be disseminated in presentations at academic national/international conferences and publication in peer-review journals.

For peer review only

Strength and limitations of this study

- We will use a non-invasive ^{13}C-breath test to construct a predictive algorithm for detecting pancreatic cancer (PC).
- PC patients with potentially curative pancreatic resection will be recruited in this study.
- The predictive algorithm may have poor ability to discriminate between PC and chronic pancreatitis patients.
- Participants' return of faecal specimens may be limited by embarrassment, hesitancy or inability of the study participants to provide a sample.

For peer review only

For peer review only - http://bmjopen.bmj.com/site/about/guidelines.xhtml

INTRODUCTION

Pancreatic cancer (PC) is the fifth leading cause of cancer related death in the UK. The UK has one of the worst survival rates in Europe, with an average life expectancy at diagnosis of just 4–6 months and 20% survival at 1 year. In addition, in the UK, only about 7% of people survive for 5 years or longer[1]**.** Since complete surgical resection remains the only potentially curative option for PC, it is crucial to identify PC at an early stage, when it is still resectable. Unfortunately, only 10–20% of patients with PC are diagnosed at a stage when curative surgery remains an option[2].

The early symptoms of PC are usually vague and include weight loss, upper abdominal pain, backache, fatigue, diarrhoea and nausea. In 93% of patients, these symptoms may have been present for up to two years before diagnosis of PC [3]. Recent studies show that around 40% of patients diagnosed with PC require three or more visits to their GP before they are referred to a specialist [4 5] thereby leading to a delay in the diagnosis. There is also an increased risk of developing PC in those with new-onset diabetes mellitus (NODM) i.e. less than three years since diagnosis [6] and as many as 25% of PC cases are believed to present with NODM [7].

Recent survey of GPs commissioned by Pancreatic Cancer Action [8] suggested that the delay between not having access to a reliable diagnostic referral pathway and a confirmed diagnosis of PC is a major barrier to the early detection of PC. As the symptoms of PC are often non-specific and unlikely to prompt further clinical investigation, there is an urgent need to have a reliable and cheap screening test that can be used to identify high-risk individuals who require urgent imaging studies such as CT scan to confirm or exclude the

For peer review only - http://bmjopen.bmj.com/site/about/guidelines.xhtml

presence of a pancreatic mass [9]. Having such a test with reliable sensitivity and negative predictive value (NPV), would enable the GP to select patients for urgent referral to further investigations of PC. A high NPV value would enable the GP to reassure the patient that they did not need further imaging studies. Our innovative approach is to use tests of pancreatic exocrine function combined as a screening test in patients presenting with the vague symptoms that may be associated with PC.

Although pancreatic exocrine insufficiency (PEI) is a well-known complication of pancreatic cancer [10 11], its presence is frequently overlooked in patients with advanced PC resulting in a decreased quality of life (QoL), malnutrition and morbidity [12]. Currently, the diagnostic tests of pancreatic exocrine function such as faecal elastase-1 (FE-1) or the ^{13}C-mixed triglyceride (^{13}C-MTG) breath test, may be used to detect PEI in patients after pancreatic surgery and chronic pancreatitis [13-16].

FE-1 test is a simple indirect and non-invasive method for assessing pancreatic secretion, which measures faecal concentrations of elastase-1, a proteolytic enzyme produced exclusively by pancreatic acinar cells, which binds to bile salts and passes through the gut with minimal degradation. FE-1 level correlate well with output of other pancreatic enzymes, such as amylase, lipase and trypsin [17 18] and have been shown to detect PEI in patients with advanced PC and following pancreatic surgery [14-16]**.**

The ^{13}C-MTG breath test assesses pancreatic lipase, it involves the ingestion of a ^{13}C-labeled mixed triglyceride (1,3-distearoyl, 2- (carboxyl-^{13}C)-octanoyl glycerol) mixed with a test meal. The ^{13}C-MTG contains a ^{13}C-labeled medium-chain fatty acid (^{13}C-octanoate) at the Sn-

For peer review only - http://bmjopen.bmj.com/site/about/guidelines.xhtml

2 position, and two long-chain fatty acids (stearic acid) at the Sn-1 and Sn-3 positions of the glycerol backbone of the triacylglycerol. The ingested ^{13}C-MTG then undergoes the lipolysis of the two-long chain fatty acids from ^{13}C-MTG by pancreatic lipase to produce free fatty acids (stearic acid and ^{13}C-octanoate) and monoacylglycerol. After the intestinal absorption, the ^{13}C-free fatty acids are oxidised in the liver producing $^{13}CO_2$, which is then released on breath. Therefore, the $^{13}CO_2$ concentration in breath reflects pancreatic lipase activity. The ^{13}C-MTG breath test can be an alternative or complementary to the FE-1 test for PEI. The ^{13}C-MTG breath test has been validated in patients with chronic pancreatitis [19] and patients with pancreatic cancer following surgical resection of the cancer [20 21], but not prior to surgery in patients with resectable PC.

Therefore, in order to improve the prognosis of patients with pancreatic cancer, it is essential to detect tumors at early stages, when they are more likely to be resectable. In this study, we will validate FE-1 test and ^{13}C-MTG breath test as potential early diagnostic biomarkers of resectable pancreatic cancer and test their performance in discriminating between patients with PC and healthy subjects. These tests can provide a non-invasive risk stratification tool to identify patients at risk of having PC when they present with NODM, weight loss or vague upper GI symptoms. Furthermore, this will provide a rational approach to patients identified to have PEI to be selected to have pancreatic imaging such as computed tomography (CT) scan to confirm the presence of PC and having a pancreatic resection earlier (Figure 1). In addition, we will also be identifying other potential biomarkers of PC through unbiased multiomics analysis of blood samples (proteomics and metabolomics). The resulting data will be compared to the healthy control group and patients with chronic pancreatitis aiming to identify signals that are distinct to PC.

For peer review only - http://bmjopen.bmj.com/site/about/guidelines.xhtml

METHODS AND ANALYSIS

Main centre

NIHR Southampton Biomedical Research Centre, Southampton Centre for Biomedical Research, University Hospital Southampton NHS Foundation Trust, Southampton, UK.

Dates of the study

From 16th March 2021 to 16th March 2022.

Design

This is a single cross-sectional pilot study of patients with PC, chronic pancreatitis and healthy controls, matched for age and sex. The primary objective is to determine if ^{13}C-mixed triglyceride (^{13}C-MTG) breath test and faecal elastase-1 (FE-1) concentrations combined can discriminate between the groups. Patients with PC will be recruited at the pre-operative stage within two weeks of initial diagnosis. The secondary objective of this study is the analysis of multiomic (proteomic and metabolomic) profiles and comparison between the groups.

Inclusion criteria

At the University Hospital Southampton NHS Foundation Trust, on average, 120 patients with PC are treated with surgery while 350 patients with PC are treated with chemotherapy every year. Male and female participants aged from 30-85 within the Wessex pancreatic cancer catchment area will be suitable for inclusion. In the cancer cohort, patients with a diagnosis of resectable pancreatic ductal adenocarcinoma (PDAC) (stage 1-2) will be included. Patients with chronic pancreatitis and healthy subjects will also be recruited as control groups and matched for age and sex to the cases of PDAC.

For peer review only - http://bmjopen.bmj.com/site/about/guidelines.xhtml

Exclusion criteria

Patients and controls with autoimmune or other chronic inflammatory conditions, or those who have previously had pancreatic disease or liver dysfunction will be excluded.

For peer review only

For peer review only - http://bmjopen.bmj.com/site/about/guidelines.xhtml

Intervention(s) or method

Each participant will be given a participant information sheet explaining the research and will sign an informed consent form. Participants are required to fast overnight for at least 12 hours prior to undergoing the ^{13}C-mixed triglyceride (^{13}C-MTG). At the Clinical Research Facility (CRF), full infection control precautions (related to COVID-19 pandemic) will be adhered to throughout the visit. Participants will have their temperature checked on arrival to ensure they are not pyrexial as part of screening for Covid-19, which will exclude them from the study. Anthropometric measurements (height, weight and waist circumference) of the participants will be recorded. Baseline blood samples will be taken prior to undergoing the ^{13}C-MTG breath test. The data collected will include the following: Full blood count (FBC), Urea and Electrolyte blood test (U&E), Liver function tests (AST, ALT, ALP, albumin and GGT) /clotting studies, glycated hemoglobulin (HbA1c %), Lipid profile, Vitamins and micronutrients, Hyaluronic acid, Procollagen III amino terminal peptide (PIINP) and Tissue inhibitor of metalloproteinase-1 (TIMP-1). These tests will be analysed immediately after the blood has been drawn from the participants by the chemical pathology service laboratory at Southampton General Hospital. Further blood samples will be processed and stored for multiomics analysis. Participants will also be asked to provide spot stool samples for faecal elastase-1 (FE-1) concentrations by using a commercially available ELISA kit. Results of the FE-1 test will be expressed as µg/g of stool.

For peer review only

For peer review only - http://bmjopen.bmj.com/site/about/guidelines.xhtml

^{13}C-MTG breath test protocol

Each participant will be asked to provide 2 baseline breath samples by blowing into 2 × 10ml exetainer tubes through a straw while wearing a facemask. After that, participants will be given an oral dose of 250mg of 2-[^{13}C]-octanoyl-1,3-distearin (^{13}C-MTG -Cambridge Isotope Laboratories, Andover, MA, USA) together with a solid test meal consisting of a crispbread or other gluten-free alternative, and 200ml of water. Post-prandial breath samples will be then collected every 30mins for 4 hours. No food or drinks except for water will be allowed during the duration of the breath test study. The collected breath samples will be analysed for the ratio of $^{13}CO_2$ to $^{12}CO_2$ using a Continuous Flow Isotope Ratio Mass Spectrometer (CF-IRMS SERCON Ltd, Crewe, UK) at the Southampton Centre for Biomedical Research. The increase in $^{13}CO_2$ content with regard to the baseline value of the initial breath sample is expressed as atom % excess and we will express the results of the ^{13}C-MTG breath test as the cumulative percentage of ^{13}C-label recovered on breath as $^{13}CO_2$ over 4 hours (cPDR over 4 hours).

Primary Outcome: Prediction of PC using a logistic model with measurement of FE-1 concentration expressed as µg/g of stool and ^{13}C-MTG breath test expressed as cPDR over 4hrs as covariates.

For peer review only

Statistical analysis and data analysis plans

Sample size calculation

The sample size calculation for the proposed study is based on a previous study assessing pancreatic exocrine function using a ^{13}C-triglyceride breath test in healthy subjects and patients with a localised pancreatic mass [13]. In this study, they used the ^{13}C-trioctanoin breath test to assess pancreatic exocrine function in 14 patients with a localised pancreatic mass and 5 healthy control subjects. The results of the study showed means ± standard deviation (SD) of the recovery of $^{13}CO_2$ over 3hrs after undergoing the ^{13}C-trioctanoin breath test of 42% ± 3.4% for the healthy controls and 24.2% ± 10.5% for patients with a localised pancreatic mass with an effect size or mean difference between both groups of 17.8% and a within group SD of 9.1% (based on the SD estimates of 3.4% and 10.5% for each group respectively).

Using data from this study, we calculated that a sample size of 25 subjects in each group (patients with PC, patients with chronic pancreatitis and healthy control subjects) will give 100% power to detect a difference of 17.8% between patients with PC and healthy control group assuming a standard deviation of 10.5% in patients with PC at a significance level of 5% using a two-tailed test. The power calculation was carried out using IBM SPSS SamplePower version 3.

For peer review only - http://bmjopen.bmj.com/site/about/guidelines.xhtml

Statistical tests

Data will be presented as mean + SD, median + IQR when appropriate and 95% CI. Comparison of results of FE-1 and the ^{13}C-MTG breath test between each group will be evaluated by employing either the one way analysis of variance test (if the variables are normally distributed and parametric validity conditions are fulfilled) or the Mann Whitney U-test or Kruskal-Wallis test (if the variables are not normally distributed and non-parametric validity conditions are fulfilled). A multi-variable logistic regression analysis will be performed in order to determine the independent factors that are significantly associated with the presence of PDAC and logistic regression models will be constructed based on the identified independent factors. Furthermore, multi-variable regression analysis will be used to determine the optimal algorithm that combines the results of FE-1 test and ^{13}C-MTG breath test. We will also test whether potential biomarkers of PC identified by unbiased omics analysis improve the prediction of PDAC. A Receiver operating characteristic (ROC) curve will be constructed to evaluate the diagnostic accuracy (sensitivity, specificity, positive and negative predictive value, receiver operator curve analysis) of the algorithm for PDAC. Furthermore, the optimal cut off point for the algorithm to identify patients with PC will be estimated using the Youden index. A two-sided p value of <0.05 will be considered to indicate statistical significance. All analysis will be performed using SPSS (IBM, V.25.0) or Stata (Statacorp, v16.0).

Patient and public involvement statement

Patients, healthy subjects and the public were not involved in the study design, recruitment and conduct. We do not plan to inform the result to the study participants unless they apply for it.

For peer review only

For peer review only - http://bmjopen.bmj.com/site/about/guidelines.xhtml

ETHICS AND DISSEMINATION

Ethical approval has been granted by the North of Scotland Research and Ethics Committee, reference: 20/NS/0105, 1 October 2020. This manuscript reflects the latest protocol V.7 approved 29 October 2020.

The study will be conducted according to the principles of Good Clinical Practice, General data Protection Regulation and Data Protection Act 2018 for Health and care Research. The sponsor and study team will ensure approval of the study protocol, participant information sheets, consent forms, letters to general practitioners and supporting documents by the appropriate regulatory body and research and ethics committee prior to participant recruitment. Documents will be stored securely with restricted access for at least 10 years. Written, informed consent will be obtained from each patient and an identification number provided. Any published data will not contain personal identifiable data. The results of this study will be disseminated by presentation at academic national/international conferences and publication in peer-review journals.

For peer review only

Author affiliations

[1] National Institute for Health Research Southampton Biomedical Research Centre, University of Southampton and University Hospital Southampton NHS Foundation Trust, Southampton, UK.

[2] School of Human Development and Health, Faculty of Medicine, University of Southampton, Southampton, UK.

[3] University Hospital Southampton NHS Foundation Trust, Southampton, UK.

[4] Southampton Clinical Trials Unit, University Hospital Southampton NHS Foundation Trust, Southampton, UK.

Acknowledgements: The authors will like to thank all the patients and healthy subjects who participated in this study.

Authors' contributions: ZH and PA have designed the study concept and formulated the initial idea; ZH is the Chief investigator and responsible for protocol review. DM and PA wrote the initial protocol. CB, PA, SW and ZH were involved in statistical analysis plan. DM and ZH was responsible for organising the study operation requirements, identifying and discussion with eligible participants. DM is responsible for patient recruitment and consent. DM and PA prepared the test meal and obtained the samples from the participants, VG is sorting the standard operating procedures (SOPs) and logistics with biobanking and sample process. PA is responsible for the analysis of the breath samples and the interpretation of the results from the breath test. JW is manging the study team and logistics related to clinical trial involvement. PA, DM, CB, SW, VG, JW and ZH were involved in drafting of the protocol.

Funding: This work is supported by Cancer Research UK (grant number C45617/A29908). DM partly funded by NIHR-BRC fellowship grant number NIHR-INF-0953.

Competing interest: None.

Patient consent for publication: Not required.

Provenance and peer review: Not commissioned; externally peer reviewed.

ORCID IDs

Paul R Afolabi https://orcid.org/0000-0002-0553-1578

Zaed Z Hamady https://orcid.org/0000-0002-4591-5226

For peer review only

References:

1. Allemani C, Matsuda T, Di Carlo V, et al. Global surveillance of trends in cancer survival 2000–14 (CONCORD-3): analysis of individual records for 37 513 025 patients diagnosed with one of 18 cancers from 322 population-based registries in 71 countries. *The Lancet* 2018;391(10125):1023-75. doi: 10.1016/s0140-6736(17)33326-3
2. Roberts KJ, Prasad P, Steele Y, et al. A reduced time to surgery within a 'fast track' pathway for periampullary malignancy is associated with an increased rate of pancreatoduodenectomy. *HPB (Oxford)* 2017;19(8):713-20. doi: 10.1016/j.hpb.2017.04.011 [published Online First: 2017/06/02]
3. Keane MG, Horsfall L, Rait G, et al. A case-control study comparing the incidence of early symptoms in pancreatic and biliary tract cancer. *Bmj Open* 2014;4(11) doi: ARTN e005720

10.1136/bmjopen-2014-005720

4. Lyratzopoulos G, Abel GA, McPhail S, et al. Measures of promptness of cancer diagnosis in primary care: secondary analysis of national audit data on patients with 18 common and rarer cancers. *Br J Cancer* 2013;108(3):686-90. doi: 10.1038/bjc.2013.1 [published Online First: 2013/02/09]
5. Lyratzopoulos G, Neal RD, Barbiere JM, et al. Variation in number of general practitioner consultations before hospital referral for cancer: findings from the 2010 National Cancer Patient Experience Survey in England. *Lancet Oncol* 2012;13(4):353-65. doi: 10.1016/S1470-2045(12)70041-4 [published Online First: 2012/03/01]
6. Chen W, Butler RK, Lustigova E, et al. Validation of the Enriching New-Onset Diabetes for Pancreatic Cancer Model in a Diverse and Integrated Healthcare Setting. *Dig Dis Sci* 2021;66(1):78-87. doi: 10.1007/s10620-020-06139-z [published Online First: 2020/03/01]
7. Chari ST, Leibson CL, Rabe KG, et al. Pancreatic cancer-associated diabetes mellitus: prevalence and temporal association with diagnosis of cancer. *Gastroenterology* 2008;134(1):95-101. doi: 10.1053/j.gastro.2007.10.040 [published Online First: 2007/12/07]
8. Action. PC. GPs dub it the 'silent killer' yet just 3% say they are fully confident in recognising symptoms. 2016 [Available from: pancreaticcanceraction.org/news/gps-dub-it-the-silent-killer-yet-just-3-say-they-are-fully-confident-in-recognising-symptoms/.
9. Collins GS, Altman DG. Identifying patients with undetected pancreatic cancer in primary care: an independent and external validation of QCancer((R)) (Pancreas). *Br J Gen Pract* 2013;63(614):e636-42. doi: 10.3399/bjgp13X671623 [published Online First: 2013/09/04]
10. Bartel MJ, Asbun H, Stauffer J, et al. Pancreatic exocrine insufficiency in pancreatic cancer: A review of the literature. *Dig Liver Dis* 2015;47(12):1013-20. doi: 10.1016/j.dld.2015.06.015 [published Online First: 2015/07/28]
11. Vujasinovic M, Valente R, Del Chiaro M, et al. Pancreatic Exocrine Insufficiency in Pancreatic Cancer. *Nutrients* 2017;9(3) doi: 10.3390/nu9030183 [published Online First: 2017/03/01]
12. Perez MM, Newcomer AD, Moertel CG, et al. Assessment of weight loss, food intake, fat metabolism, malabsorption, and treatment of pancreatic insufficiency in pancreatic cancer. *Cancer* 1983;52(2):346-52. doi: 10.1002/1097-0142(19830715)52:2<346::aid-cncr2820520228>3.0.co;2-z [published Online First: 1983/07/15]

For peer review only - http://bmjopen.bmj.com/site/about/guidelines.xhtml

13. Kato H, Nakao A, Kishimoto W, et al. 13C-labeled trioctanoin breath test for exocrine pancreatic function test in patients after pancreatoduodenectomy. *Am J Gastroenterol* 1993;88(1):64-9. [published Online First: 1993/01/01]

14. Matsumoto J, Traverso LW. Exocrine function following the whipple operation as assessed by stool elastase. *J Gastrointest Surg* 2006;10(9):1225-9. doi: 10.1016/j.gassur.2006.08.001 [published Online First: 2006/11/23]

15. Sikkens EC, Cahen DL, de Wit J, et al. Prospective assessment of the influence of pancreatic cancer resection on exocrine pancreatic function. *Br J Surg* 2014;101(2):109-13. doi: 10.1002/bjs.9342 [published Online First: 2013/12/18]

16. Sikkens EC, Cahen DL, de Wit J, et al. A prospective assessment of the natural course of the exocrine pancreatic function in patients with a pancreatic head tumor. *J Clin Gastroenterol* 2014;48(5):e43-6. doi: 10.1097/MCG.0b013e31829f56e7 [published Online First: 2014/04/11]

17. Levy P, Barthet M, Mollard BR, et al. Estimation of the prevalence and incidence of chronic pancreatitis and its complications. *Gastroenterol Clin Biol* 2006;30(6-7):838-44. doi: 10.1016/s0399-8320(06)73330-9 [published Online First: 2006/08/04]

18. Van de Vijver E, Desager K, Mulberg AE, et al. Treatment of infants and toddlers with cystic fibrosis-related pancreatic insufficiency and fat malabsorption with pancrelipase MT. *J Pediatr Gastroenterol Nutr* 2011;53(1):61-4. doi: 10.1097/MPG.0b013e31820e208e [published Online First: 2011/06/23]

19. Gonzalez-Sanchez V, Amrani R, Gonzalez V, et al. Diagnosis of exocrine pancreatic insufficiency in chronic pancreatitis: (13)C-Mixed Triglyceride Breath Test versus Fecal Elastase. *Pancreatology* 2017;17(4):580-85. doi: 10.1016/j.pan.2017.03.002 [published Online First: 2017/03/16]

20. Dominguez-Munoz JE, Iglesias-Garcia J, Vilarino-Insua M, et al. 13C-mixed triglyceride breath test to assess oral enzyme substitution therapy in patients with chronic pancreatitis. *Clin Gastroenterol Hepatol* 2007;5(4):484-8. doi: 10.1016/j.cgh.2007.01.004 [published Online First: 2007/04/21]

21. Muniz CK, dos Santos JS, Pfrimer K, et al. Nutritional status, fecal elastase-1, and 13C-labeled mixed triglyceride breath test in the long-term after pancreaticoduodenectomy. *Pancreas* 2014;43(3):445-50. doi: 10.1097/MPA.0000000000000048 [published Online First: 2014/03/14]

For peer review only

For peer review only - http://bmjopen.bmj.com/site/about/guidelines.xhtml

399 **Figure 1** Proposed diagnostic tool consisting of faecal elastase-1 test and the ^{13}C-mixed triglyceride breath test to screen for pancreatic exocrine dysfunction in patients 'at-risk' of pancreatic cancer.

402

403

404

For peer review only

For peer review only - http://bmjopen.bmj.com/site/about/guidelines.xhtml

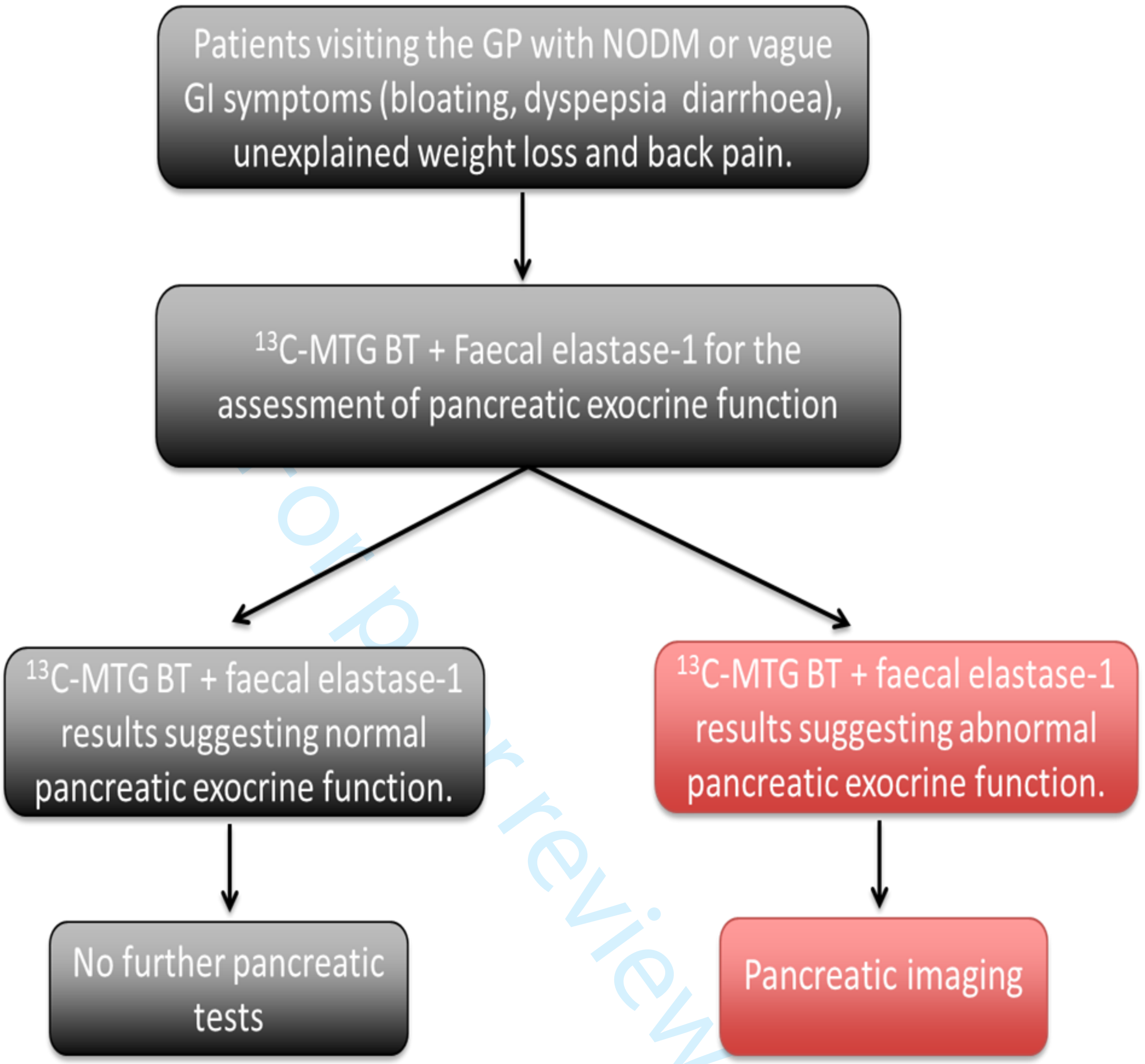

Abbreviations: NODM- New Onset Diabetes Mellitus, ^{13}C-MTG BT- ^{13}C-mixed triglyceride breath test.

For peer review only

TRIPOD Checklist: Prediction Model Development

Section/Topic	Item	Checklist Item	Page
**Title and abstract**			
Title	1	Identify the study as developing and/or validating a multivariable prediction model, the target population, and the outcome to be predicted.	Page 1, lines 11-14
Abstract	2	Provide a summary of objectives, study design, setting, participants, sample size, predictors, outcome, statistical analysis, results, and conclusions.	Page 2, lines 22-43.
**Introduction**			
Background and objectives	3a	Explain the medical context (including whether diagnostic or prognostic) and rationale for developing or validating the multivariable prediction model, including references to existing models.	Pages 4-6, lines 57-130.
	3b	Specify the objectives, including whether the study describes the development or validation of the model or both.	Page 6, lines 124-130.
**Methods**			
Source of data	4a	Describe the study design or source of data (e.g., randomized trial, cohort, or registry data), separately for the development and validation data sets, if applicable.	Page 8, lines 145-152
	4b	Specify the key study dates, including start of accrual; end of accrual; and, if applicable, end of follow-up.	Page 8, lines 142-143.
Participants	5a	Specify key elements of the study setting (e.g., primary care, secondary care, general population) including number and location of centres.	Page 8, lines 139-141.
	5b	Describe eligibility criteria for participants.	Page 8, lines 153-161.
	5c	Give details of treatments received, if relevant.	n/a
Outcome	6a	Clearly define the outcome that is predicted by the prediction model, including how and when assessed.	Page 10, lines 203-205.
	6b	Report any actions to blind assessment of the outcome to be predicted.	n/a
Predictors	7a	Clearly define all predictors used in developing or validating the multivariable prediction model, including how and when they were measured.	n/a
	7b	Report any actions to blind assessment of predictors for the outcome and other predictors.	n/a
Sample size	8	Explain how the study size was arrived at.	Page 11, lines 215-222.
Missing data	9	Describe how missing data were handled (e.g., complete-case analysis, single imputation, multiple imputation) with details of any imputation method.	n/a
Statistical analysis methods	10a	Describe how predictors were handled in the analyses.	n/a
	10b	Specify type of model, all model-building procedures (including any predictor selection), and method for internal validation.	Page 12, lines 231-249.
	10d	Specify all measures used to assess model performance and, if relevant, to compare multiple models.	n/a
Risk groups	11	Provide details on how risk groups were created, if done.	n/a
**Results**			
Participants	13a	Describe the flow of participants through the study, including the number of participants with and without the outcome and, if applicable, a summary of the follow-up time. A diagram may be helpful.	n/a
	13b	Describe the characteristics of the participants (basic demographics, clinical features, available predictors), including the number of participants with missing data for predictors and outcome.	n/a
Model development	14a	Specify the number of participants and outcome events in each analysis.	n/a
	14b	If done, report the unadjusted association between each candidate predictor and outcome.	n/a
Model specification	15a	Present the full prediction model to allow predictions for individuals (i.e., all regression coefficients, and model intercept or baseline survival at a given time point).	n/a
	15b	Explain how to the use the prediction model.	n/a
Model performance	16	Report performance measures (with CIs) for the prediction model.	n/a
**Discussion**			
Limitations	18	Discuss any limitations of the study (such as nonrepresentative sample, few events per predictor, missing data).	n/a

For peer review only

TRIPOD Checklist: Prediction Model Development

Interpretation	19b	Give an overall interpretation of the results, considering objectives, limitations, and results from similar studies, and other relevant evidence.	n/a
Implications	20	Discuss the potential clinical use of the model and implications for future research.	n/a
**Other information**			
Supplementary information	21	Provide information about the availability of supplementary resources, such as study protocol, Web calculator, and data sets.	n/a
Funding	22	Give the source of funding and the role of the funders for the present study.	Page 14, lines 303-304.

We recommend using the TRIPOD Checklist in conjunction with the TRIPOD Explanation and Elaboration document.

For peer review only
